# Anthocyanin-Rich Pigment Supplements in the Australian Online Market: Sources, Labelling Practices, and Bioactivity Claims

**DOI:** 10.3390/foods15060992

**Published:** 2026-03-11

**Authors:** Ravish Kumkum, Katherine M. Livingstone, Kathryn Aston-Mourney, Bryony A. McNeill, Leni R. Rivera

**Affiliations:** 1 Institute for Mental and Physical Health and Clinical Translation (IMPACT), School of Medicine, Deakin University, Geelong 3220, Australia; k.ravish@deakin.edu.au (R.K.); k.astonmourney@deakin.edu.au (K.A.-M.); bryony.mcneill@deakin.edu.au (B.A.M.); 2 Institute for Physical Activity and Nutrition (IPAN), School of Exercise and Nutrition Sciences, Deakin University, Geelong 3220, Australia; k.livingstone@deakin.edu.au

**Keywords:** anthocyanins, bioactive pigments, nutraceuticals, dietary supplements, health claims, polyphenols, product labelling

## Abstract

Amid growing consumer interest in the health benefits of anthocyanins, the market for anthocyanin-based dietary supplements has expanded substantially. However, data on product composition and health claims in the Australian online market remains limited. This study characterises anthocyanin-based dietary supplements available online in Australia (July–August 2024), examining product origin, botanical sources, formulation, extract concentration, delivery formats, anthocyanin content disclosure, health claims, serving sizes and pricing patterns. Among the 121 products analysed, bilberry, elderberry, and tart cherry were the most commonly used anthocyanin sources, collectively representing 47% of the market. Capsules were the dominant delivery format (86%), and reported extract concentrations varied widely, with lower ratios (1–10:1) being most prevalent. Only 18% of products disclosed anthocyanin content (0.1–36%), with bilberry supplements accounting for the majority of reported values. Health claims were present on 69% of products and varied by source, with bilberry products most frequently promoting vision health, elderberry emphasising immune support, tart cherry targeting joint and exercise recovery, and cranberry focusing on urinary tract health. Declared extract (mg per serving) ranged from 10 mg to >1500 mg. Pricing varied substantially across products (AUD 0.02–7.40 per gram; mean AUD 0.78/g), with bilberry supplements consistently among the most expensive. Overall, these findings highlight considerable variability in formulation, disclosure, and health claim practices, underscoring the need for improved transparency and evidence-aligned representation of anthocyanins as bioactive pigments in the Australian nutraceutical market.

## 1. Introduction

Anthocyanins are naturally occurring, water-soluble polyphenolic pigments responsible for the bright red-orange to blue-violet colours observed in many plant-based foods, including fruits, vegetables, stems, leaves, and flowers [1]. Structurally, anthocyanins are flavonoids derived from the flavylium ion, comprising two benzoyl rings linked by a heterocyclic ring. To date, more than 650 anthocyanin compounds have been identified in nature, with approximately 90% represented by six major anthocyanidins: malvidin, cyanidin, delphinidin, petunidin, peonidin, and pelargonidin [2]. These compounds exhibit distinct colour characteristics and are highly sensitive to environmental and processing conditions, including pH, temperature, light, oxygen exposure, copigmentation, water activity, sugar content, enzymatic activity, and food processing techniques. Anthocyanins are widely distributed in plant-based foods, particularly fruits such as berries, grapes, and plums, as well as vegetables including red cabbage, onions, and eggplant, and pigmented grains and legumes such as black rice and black soybeans [1].

Beyond their role as natural colourants, anthocyanins have been extensively studied for their potential bioactive properties, including anti-inflammatory [3,4,5], antioxidant [6,7], anti-cancer [8,9,10], immunomodulatory [11,12], anti-microbial [13], antiaging [14], cardioprotective [15], hypoglycaemic, hypolipemic [16] and neuroprotective properties [17]. Despite these reported benefits, anthocyanins—like many other phytonutrients—exhibit low bioavailability, which limits their efficient absorption and biological efficacy following consumption [18]. Consequently, various extraction and concentration techniques have been employed to maximize their benefits. The traditional solvent extraction method is widely used, along with modern methods utilising technologies such as ultrasound, supercritical fluid extraction, microwave-assisted extraction, ohmic heating, cold extraction, pulsed electric fields, enzyme-assisted methods, and combinations of these techniques under modified temperature and pressure conditions [19]. Following extraction, the extracts are in some cases purified further to remove components other than the target bioactive compounds with an aim to concentrate them using techniques such as chromatographic methods and membrane separation [19]. However, supplements available on the market do not directly specify what method of extraction was employed but, in some cases, mention the ratio or percentage of anthocyanin content. Some novel methods have also been patented and commercially exploited to extract and capture these bioactive compounds from plants particularly in food, pharmaceutical/therapeutic and cosmetic industries reflecting the growing demand for anthocyanins as concentrated pigment-derived bioactive compounds.

The increasing availability of anthocyanin-rich foods, together with advances in extraction technologies, has contributed to the expansion of anthocyanin-based supplements derived from a wide range of plant sources. According to recent industry market intelligence forecasts, the global anthocyanin market is valued at approximately USD 415 million in 2025 and is projected to reach USD 690 million by 2035, corresponding to a compound annual growth rate of 5.2% [20]. Demand for anthocyanin-containing products is primarily driven by the food and beverage sector (48%), followed by pharmaceutical, cosmetic, and nutraceutical applications, with the nutraceutical segment expected to grow at a CAGR of 6.3% as interest in polyphenol-rich products for cardiovascular, cognitive, and ocular health continues to increase [20]. This projected market expansion is consistent with an expanding body of literature highlighting the efficacy of these specific health benefits [21,22,23].

This trend is particularly beneficial in regions where access to fresh anthocyanin-rich foods, such as berries, may be limited due to climatic, seasonal, or economic constraints. These products are commonly consumed for reasons including convenience, reduced sugar intake compared to whole-food alternatives, limited availability of specific foods, and individual dietary preferences. In addition, available evidence suggests that anthocyanins are generally well tolerated, and human and animal intervention studies have not identified any major safety concerns at commonly studied doses [24,25]. For example, recent toxicological assessments of purified anthocyanins in animal models administering oral cyanidin reported an acute median lethal dose (LD50) exceeding 300 mg/kg/day and established a 28-day subacute No Observed Adverse Effect Level (NOAEL) of 30 mg/kg/day [26]. Furthermore, authoritative safety evaluations by the Joint FAO/WHO Expert Committee on Food Additives have established an acceptable daily intake (ADI) of 2.5 mg/kg per day for anthocyanins from grape-skin extracts; however, this ADI does not apply to anthocyanins in general [27]. Accordingly, while anthocyanins are widely considered to have a favourable safety profile, extrapolation to high-dose or long-term supplementation should be made cautiously and supported by product-specific composition data and, ideally, independent verification.

A statistical report published in 2021 on anthocyanin-based products in the Chinese market indicated that a substantial proportion of major health-related product brands were of Australian origin, with Australian products ranking second in number after those from China [28]. Despite this strong international presence, comparable data characterising anthocyanin-containing supplements within the Australian market remain limited. This lack of systematically reported information restricts transparent comparison of product composition, sources, and claimed bioactivity, thereby limiting informed interpretation by researchers, health professionals, and manufacturers. To our knowledge, this study provides the first systematic characterisation of anthocyanin-based dietary supplements in the Australian online market (July–August 2024), addressing four research objectives: (i) to characterize the origin, botanical sources, and delivery formats of these supplements; (ii) to evaluate the extent and consistency of extract concentration and anthocyanin content disclosure on product labels; (iii) to assess the nature and frequency of health claims in relation to available scientific evidence; and (iv) to examine pricing patterns across different anthocyanin sources and formulations. By providing an overview of current market practices, this work aims to improve understanding of how anthocyanin pigments are represented and standardised in commercial supplements, supporting future research, product development, and application of anthocyanins as bioactive food pigments.

## 2. Materials and Methods

Data were collected from two major online retail platforms, iHerb and Amazon Australia, supplemented by targeted Google searches conducted between July and August 2024. To approximate typical consumer browsing behaviour, searches were performed using the platforms default ranking/sorting settings (e.g., “Relevance”) at the time of capture. On iHerb, two searches were performed using the keywords “anthocyanin” and “berry,” with filters applied for category (“supplements”), product form (“pill”), life stage (“adults”), and formulation (“single ingredient”). A similar approach was used on Amazon, applying the “supplements” filter, which included tablets and capsules. Google searches used predefined keyword combinations (e.g., “anthocyanin supplements Australia”, “bilberry anthocyanin supplement Australia”, elderberry anthocyanin supplement Australia”). Only organic searches were screened; sponsored placements and paid advertisements were excluded.

**Figure 1:**
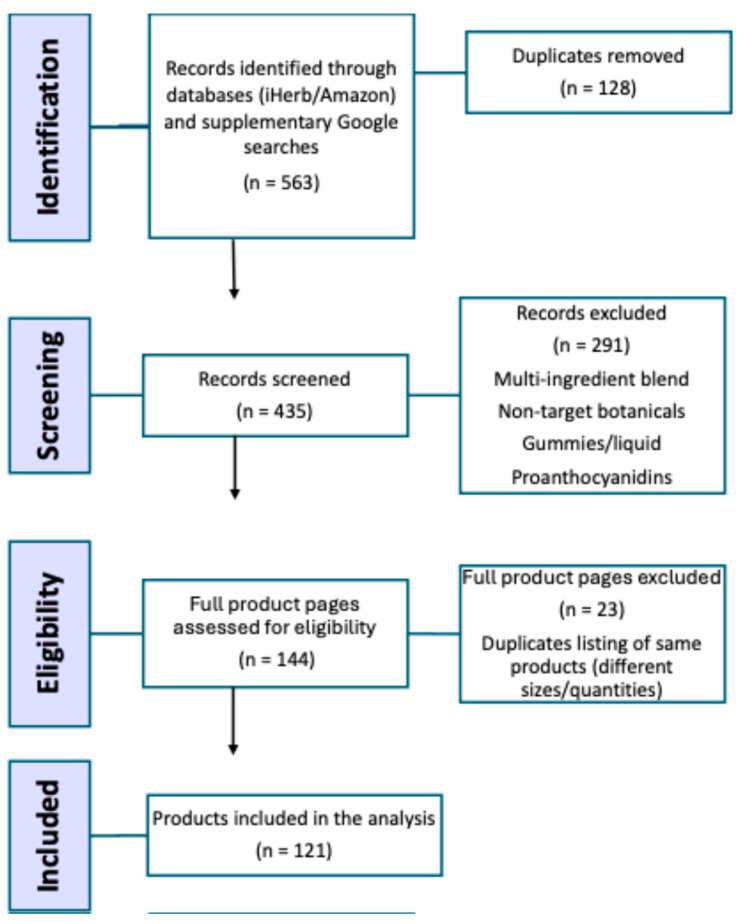
PRISMA-style flow diagram of product identification, screening, eligibility assessment, and inclusion for anthocyanin-based dietary supplements sourced from iHerb and Amazon Australia, supplemented by Google searches (July–August 2024).

Products were eligible if they were derived from anthocyanin-rich plant sources identified in the scientific literature and were formulated as single-ingredient supplements (Table 1). Multi-ingredient blends, non-target botanicals, and non-eligible formats were excluded, and discounts were not factored into the analysis. All non-target ingredients were recorded under ‘other ingredients’; exclusion applied only when they were marketed as co-primary actives. Records were screened sequentially and screening ceased when additional pages yielded no new eligible single-ingredient products (saturation stopping rule). After initial screening (*n* = 144), duplicate listings of the same product (e.g., different pack sizes/quantities) were removed, resulting in a final dataset of 121 products (Figure 1).

For each product, data were extracted from publicly available label information and product descriptions, including country of origin, botanical source, delivery format, reported fruit extract concentration or extract ratio, declared anthocyanin content, stated health-related claims, serving size, manufacturer’s price, and the presence of any additional ingredients. Data extraction was initially performed by one trained reviewer using a predefined, standardised protocol (including unit harmonisation rules and pre-specified criteria for claim categorisation). The extracted dataset was then independently cross-checked by a second reviewer, and discrepancies (<5% of records) were resolved by re-checking the original product listing and reaching consensus. To facilitate comparison across products and sources, variables such as fruit extract concentration, anthocyanin content, extract per serving, and unit price were grouped into predefined ranges. Where anthocyanin content was reported using different units or formats, values were manually standardised and, where possible, converted to percentage content to enable comparison across products. When anthocyanins were reported in mg, values were converted to standardised anthocyanin % per serving using the declared extract per serving (mg) as the denominator (see Supplementary Material for worked examples). Capsule fill mass was not used because it was rarely disclosed.

$$\text{Standardised Anthocyanins}\left(\%\right)\;\text{per serving}=\frac{\text{Declared Anthocyanin content}\left(\text{mg per serving}\right)}{\text{Declared extract}\left(\text{mg per serving}\right)}\times 100$$

When anthocyanins were reported per capsule or tablet, values were converted to declared extract per serving (mg). Percentage disclosures were retained as reported. Products without anthocyanin content disclosure were excluded from % anthocyanin analyses.

Data analysis and graphical visualisation were conducted using Microsoft Excel, including the use of pivot tables for data aggregation, summarisation, and comparative analysis.

## 3. Results

### 3.1. Country of Origin

**Figure 2:**
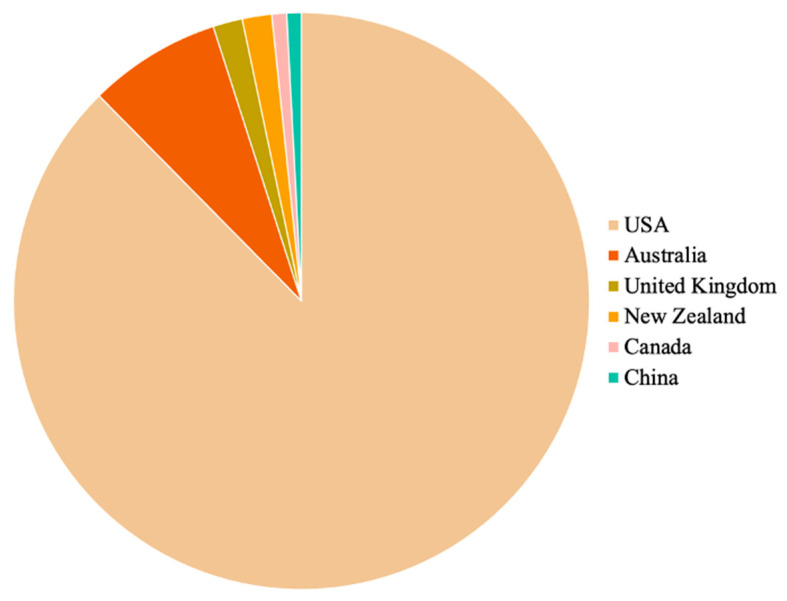
The pie chart illustrates the proportion of supplement products sourced from different countries. The majority of the products originated from the USA (87.6%), followed by Australia (7.4%). The United Kingdom and New Zealand each contributed 1.7%, while Canada and China each contributed 0.8%.

The country of origin of anthocyanin-based supplements available in the Australian online market is presented in Figure 2. For the purposes of this analysis, “origin” refers to the country in which the company is headquartered, rather than the location of product manufacture. The majority of supplements originated from companies based in the United States (106 of 121 products), followed by Australia (9 products). The United Kingdom and New Zealand each accounted for two products, while Canada and China were represented by a single product each, indicating a minimal share of the market.

### 3.2. Main Sources of Anthocyanins and Their Delivery Formats

**Figure 3:**
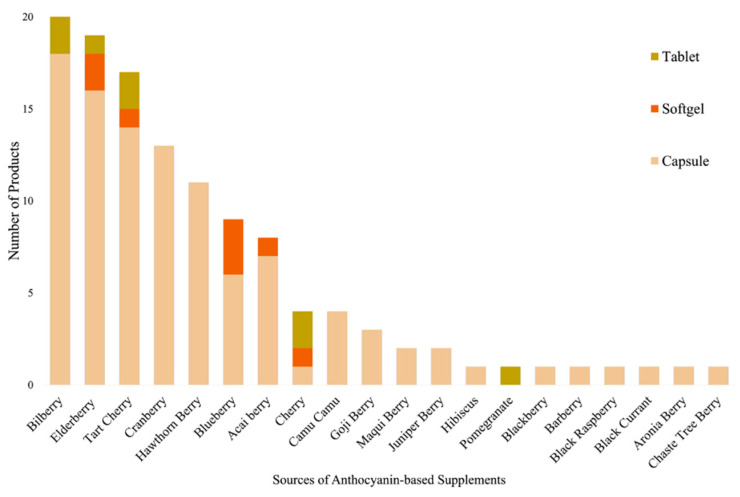
Distribution of plant sources and delivery formats for anthocyanin-based supplements in the Australian online market. Bilberry, elderberry, and tart cherry were the most common sources, while several other berries contributed in smaller proportions. Capsules were the dominant delivery format, followed by tablets and softgels.

Anthocyanin-based supplements were derived from a wide range of natural plant sources, predominantly fruits rich in anthocyanins. Analysis of the 121 supplements identified diverse botanical origins, as illustrated in Figure 3. Bilberry was the most commonly used source (17.4%), followed by elderberry (15.7%), tart cherry (14.0%), and cranberry (10.7%). Other frequently utilised sources included hawthorn berry (9.1%), blueberry (7.4%), and acai berry (6.6%). Less commonly represented sources such as camu camu (3.3%) and goji berry (2.5%) also contributed to the overall diversity. Rare sources (≤1.7%) included maqui berry, juniper berry, hibiscus, pomegranate, blackberry, barberry, black raspberry, black currant, aronia berry, and chaste tree berry. The supplements were available in several delivery formats where capsules were the predominant form, accounting for 104 of the 121 products, while eight products were formulated as softgels and nine were available as tablets.

### 3.3. Main Concentration of Fruit Extracts

The concentration (plant-to-extract ratio) of fruit extracts in anthocyanin-based supplements indicates the amount of fresh fruit equivalent delivered per serving. For example, a 4:1 extract in a 500 mg serving corresponds to 2000 mg fresh fruit equivalent, whereas a 100:1 extract represents 100 times the weight of the fresh fruit source. Among the 121 anthocyanin-based supplements, 70 products (57.9%) did not report an extract ratio, limiting assessment of the actual fruit equivalent content. The remaining 51 products (42.1%) disclosed extract concentrations and were classified into low (1–10:1), moderate (11–50:1), and high (>50:1).

Low concentrations, ranging from 1–10:1, accounted for 25.6% of all supplements. The most common extract ratios were 4:1 and 10:1, primarily found in bilberry, elderberry, tart cherry, acai berry, cranberry, and hawthorn berry. Other concentrations, such as 7.5:1 in acai berry and hawthorn berry, 5:1 in aronia berry, acai berry, black raspberry, and tart cherry, and lastly 9:1 in sweet cherry were observed.

Moderate concentrations, between 11 and 50:1, made up 10.7% of the supplements. Extract ratios such as 12:1 in hawthorn berry and goji berry, 15:1 in elderberry, 20:1 in elderberry, and 25:1 in tart cherry were observed. The most frequently reported in this category was 50:1, found in tart cherry, blueberry, and cranberry. Additionally, a concentration of 36:1 was reported in blueberry, cherry, and cranberry.

High concentrations, above 50:1, were the least common, representing 5.8% of supplements. The highest concentration observed was 100:1, exclusively in bilberry supplements. Other highly concentrated extracts included 55:1 in blueberry, 64:1 in elderberry, 75:1 in elderberry, and 52:1 in tart cherry.

### 3.4. Levels of Anthocyanins in Percentage

A total of 22 products reported anthocyanin content, ranging from 0.1% to 36%, with both the lowest and highest values found in bilberry products. These anthocyanin levels were classified into three concentration ranges based on tertiles: 0–12%, 13–24%, and 25–36%.

Only five fruit sources including bilberry, blueberry, cherry, elderberry, and tart cherry reported anthocyanin percentages. Bilberry-based supplements had the highest representation, with 13 products disclosing this information. Among them, six contained 25% anthocyanins, four had 36%, and the remaining three reported concentrations of 3%, 12.5%, and 32%, respectively.

Tart cherry was the second most frequently reported source, with five products reporting percentages of 0.1%, 0.3%, 0.8%, 3%, and 10%, each in a single product. Blueberry-based supplements reported percentages of 1.5% and 2.5% of anthocyanins, each in one product, while elderberry and cherry supplements each reported a single concentration of 14% and 0.8% of anthocyanins, respectively.

### 3.5. Declared Health Claims

An analysis of 121 anthocyanin-based supplements revealed that 37 products did not report any health claims. For the remaining 84 products, health claims were categorized under eight key health benefit classifications: (i) Antioxidant and Cellular Health, (ii) Cardiovascular Health, (iii) Immune Support, (iv) Joint, Muscle and Exercise Recovery, (v) Memory and Cognition, (vi) Urinary and Reproductive Health, (vii) Vision Health, and (viii) Other Metabolic Health Support.

Bilberry supplements presented the highest number of health claims among all analysed products, with 71.4% mentioning health claims. Vision health emerged as the most frequently highlighted category reported by 86.7% of products, followed by antioxidant and cellular health at 26.7%, cardiovascular health at 20.0%, and other metabolic health support at 13.3%. Under vision health, specific claims included “promotes healthy vision,” “supports eye health and eye function,” and “helps eyes adapt to the dark,” “maintains healthy eyesight and eye comfort,” and “helps with nighttime visual acuity.” Some products emphasized bilberry’s ability to enhance ocular circulation, reduce eye strain, and neutralize free radicals to protect delicate ocular tissues with the statements “offers antioxidant support to neutralize free radicals and support ocular capillaries” and “provides antioxidant nutrition for the eyes.” Three products highlighted cardiovascular health benefits, including claims such as “supports healthy blood flow,” “maintains blood capillary health,” and lastly one product had a unique claim “reduces symptoms of mild varicose veins and haemorrhoids.” Other metabolic health support claims included those for liver health, and joint health. For elderberry-based supplements, 68.4% reported health claims, with 69.2% of them claiming immune support, followed by 23.1% for antioxidant and cellular health, and 15.4% for cardiovascular health. Specific immune support claims included “supports upper respiratory tract health,” “helps soothe the throat,” and “helps reduce common cold symptoms.” Among tart cherry supplements, 70.6% included health claims across six categories. Joint, Muscle, and Exercise Recovery was the most frequently cited category, accounting for 66.7% of claims, followed by Antioxidant and Cellular Health at 50% and Cardiovascular Health at 16.7%. Urinary and Reproductive Health, Immune Support, Vision Health, and Memory and Cognition were each reported in 8.3% of products. Specific Joint, Muscle, and Exercise Recovery claims included “joint health and mobility,” “supports joint integrity, movement and flexibility,” “benefits joint health by strengthening collagen structures in connective tissues,” “promotes healthy uric acid balance,” “supports joint flexibility and comfort,” and “aids recovery after exercise.” Claims for urinary and reproductive health included support for urinary tract health and uric acid maintenance. Other claims highlighted antioxidant and anti-inflammatory properties, heart health, circulation, eye health, and cognitive support. For cranberry supplements, 88.2% included health claims, primarily related to Urinary and Reproductive Health by 76.5% of products and 11.8% for Antioxidant and Cellular Health. Some specific urinary tract health claims included “may help reduce the risk of recurrent urinary tract infection in healthy women,” “supports urinary tract health and function, including bladder and kidney health,” and “provides relief from symptoms of medically diagnosed cystitis such as itching and frequent, burning urination.” Health claims were identified in 50% of acai berry supplements, with 75% highlighting antioxidant activity, while 25% mentioned support for immune system health. All sweet cherry products declared health claims, with antioxidant activity highlighted in all products, while two products included claims for joint health which stated “supports joint structures and uric acid metabolism” similar to tart cherry. Health claims were identified in 55.6% of blueberry supplements, with two products highlighting vision health, two focused on antioxidant activity, and one included unique claims for promoting DNA function and supporting memory and cognition. Hawthorn berry supplements showed health claims in 45.5% of products, with all claims focused on cardiovascular health, including statements such as “supports circulation,” “maintains blood pressure levels,” and “supports healthy lipid levels”.

Other fruit extracts, including camu camu, goji berry, maqui berry, black currant, black raspberry, chaste tree berry, juniper berry, and pomegranate, demonstrated limited health claims. Antioxidant activity and immune support were noted for camu camu and maqui berry. Goji berry was associated with vision health, chaste tree berry with female reproductive health, juniper berry with digestive health, and pomegranate with promoting DNA function.

### 3.6. Amount of Extract per Servings

**Figure 4:**
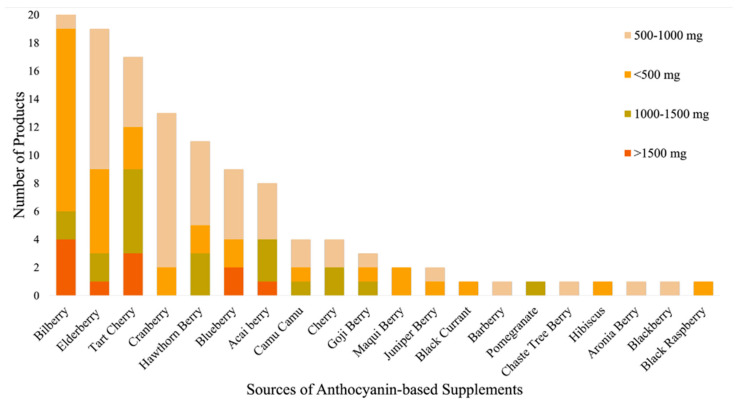
Distribution of anthocyanin-based supplement sources based on fruit extract present per serving. The majority of products fall within the 500–1000 mg range (43.8%), followed by <500 mg (29.8%), 1000–1500 mg (17.4%), and >1500 mg (9.1%).

The declared extract (mg per serving) varied widely across different fruit sources, as summarised in Figure 4. These values were categorised into four ranges based on the declared extract (mg per serving) derived from the manufacturer’s recommended serving size: <500 mg, 500–1000 mg, 1000–1500 mg, and >1500 mg. Among the sources analysed, bilberry supplements exhibited the most diverse serving sizes, with 61.9% of products having <500 mg, with 9.5% each in the 500–1000 mg and 1000–1500 mg categories and 19% exceeding 1500 mg, with the highest being 10.5 g. It was noted that to achieve higher serving sizes, two or more capsules were being recommended. Elderberry supplements displayed a broad range of serving sizes, predominantly in the 500–1000 mg range at 52.6%, with smaller proportions below 500 mg and exceeding 1500 mg. Tart cherry supplements were mostly concentrated in the 1000–1500 mg range at 35.3%, followed by 500–1000 mg at 29.4%. Cranberry and hawthorn berry supplements largely fell within the 500–1000 mg range at 84.6% and 54.5%, respectively, with none exceeding 1000 mg. Blueberry supplements showed variability, with 55.6% in the 500–1000 mg range and 22.2% exceeding 1500 mg. Acai berry supplements were primarily in the 500–1000 mg and 1000–1500 mg ranges, with none below 500 mg. Camu camu and cherry supplements were mostly in the 500–1000 mg range at 50.0%, with none exceeding 1500 mg. Less common sources like juniper berry, maqui berry, blackberry, barberry, and aronia berry were primarily below 500 mg, while goji berry and pomegranate showed greater variation across higher ranges.

### 3.7. Unit Price

To facilitate price comparison, the prices of anthocyanin-based supplements were classified into three distinct ranges per gram, determined by tertiles of the price distribution: low (0–2.5 AUD), mid (2.51–5.0 AUD), and high (5.1–7.5 AUD). Most products fell in the low-price range, representing 81.0% of products, predominantly elderberry, tart cherry, cranberry, bilberry, and hawthorn berry, blueberry and acai berry. Additional sources in this range, with fewer products, included aronia berry, barberry, black currant, black raspberry, blackberry, camu camu, cherry, goji berry, hibiscus, juniper berry, maqui berry, pomegranate, and chaste tree berry. The mid-price range, representing 5.8% of products, was largely dominated by bilberry, with minor contributions from cranberry and elderberry. The high-price range, representing 4.1% of products, was limited to bilberry and tart cherry.

### 3.8. Other Ingredients

The analysis of other ingredients used in anthocyanin-based supplements revealed a wide range of excipients incorporated for capsule formation, stabilization, and binding. Common capsule materials included gelatin (bovine or porcine), hypromellose (vegetable cellulose), and pullulan. Fillers and binders such as rice flour, maltodextrin, microcrystalline cellulose, and rice bran were frequently observed. Stabilizers included magnesium stearate, silica, stearic acid, and calcium silicate. Some supplements contained organic ingredients, including organic flaxseed oil, organic extra virgin olive oil, organic yellow beeswax, and organic rice extract blends. Other identified ingredients included dibasic calcium phosphate, acacia gum, bamboo extract, beet juice, and natural caramel colour. Additionally, components such as Active Enzymes (cellulose, pectinase, hemicellulose, and xylanase), black pepper extract, and gellan gum were present in certain products.

## 4. Discussion

This market analysis examined the characteristics of anthocyanin-based dietary supplements available in the Australian online market, providing insight into current product representation and market composition. The findings revealed a strong presence of U.S.-manufactured supplements, accounting for 87.6% of the products analysed, while Australian-made products comprised only 7.4%. In contrast, a study on the Chinese online market indicated that most anthocyanin-based supplements were domestically produced in China, with Australia and the United States ranking as the second and third largest suppliers, respectively [28]. However, the Chinese study included a broader range of products, encompassing dietary supplements, food products, and skincare items, whereas this study focused exclusively on dietary supplements. Given the growing demand for anthocyanin-containing nutraceuticals and the high proportion of imported products in the Australian market, characterising the sources, composition, and marketed attributes of these supplements is important. Variability in extract reporting, anthocyanin disclosure, and health-related claims highlights gaps in standardisation and regulatory clarity, which may affect interpretation of product potency and bioactivity. These findings are relevant for improving transparency and supporting the effective application of anthocyanins as bioactive compounds in food and nutraceutical formulations.

With respect to botanical sources, bilberry, elderberry, and tart cherry were the most prominent, collectively accounting for nearly half of the supplements analysed. Cranberry, hawthorn berry, and blueberry also showed substantial representation, together comprising nearly two-thirds of the total products. These fruits are recognised as rich sources of anthocyanins, particularly bilberry, blueberry, elderberry, and cherries [29,30], and have been widely associated with reported health-promoting properties such as antioxidant activity, cardiovascular support, anti-diabetic effects, immune modulation, and anti-cancer potential [2]. Their established association with anthocyanin content and bioactivity may therefore contribute to their dominant presence in the supplement market. In contrast, moderately represented sources such as acai berry, cherry, camu camu, and goji berry appear to target more specific consumer preferences or niche health applications. Other sources, including maqui berry, juniper berry, pomegranate, blackberry, black raspberry, black currant, and aronia berry, showed minimal market representation, which may reflect lower consumer awareness, limited availability, or challenges related to sourcing, processing, or cost.

The analysis of delivery formats for anthocyanin-based fruit extracts revealed a clear dominance of capsules, with softgels and tablets being less common. This preference for capsules aligns with trends observed in the dietary supplement market, where standardized formulations in capsule form are favoured for their perceived efficacy and portability. However, recently it is shown that softgels are gaining more popularity and preference over hard shelled capsules as it is perceived as easy to swallow, and fast acting [31]. In the present study, capsules were predominantly used for products containing anthocyanin extracts in powder form, whereas softgels were more commonly employed when extracts were formulated in liquid form. Tablets were used by a smaller number of manufacturers and were the least common delivery format observed. Although tablets remain the most widely used dosage form in pharmaceutical applications, capsules have been reported as one of the most acceptable and preferred delivery formats for dietary supplements, as they are generally associated with fewer issues related to taste or odour [32].

The concentration of fruit extracts in anthocyanin-based supplements is a key indicator of product potency, reflecting the relative density of bioactive compounds derived from fresh plant material. This concept is commonly described using the plant-to-extract ratio, defined as the ratio of the quantity of botanical material used to the quantity of extract obtained [33]. Within regulatory frameworks, this is referred to as the Drug-to-Extract Ratio (DER) by the European Medicines Agency (EMA) [34], while in Australia it is more commonly described as the extract ratio or phyto-equivalence [35]. In this study, extract concentrations varied substantially, ranging from 2.5:1 to 100:1, reflecting varied approaches to pigment concentration and extraction intensity across manufacturers. However, 58% of the analysed products did not disclose concentration details, highlighting a significant gap in market transparency. Although all products declared the extract (mg per serving), the availability and format of concentration descriptors was inconsistent which complicates direct comparisons across products. For example, some Australian labelled products report extract strength as equivalent to fresh fruit amount (e.g., “each film-coated tablet contains bilberry extract 166.67 mg, equivalent to 15 g fresh fruit”), whereas many USA-origin products typically report an extract ratio (e.g., 10:1 or 12:1). While both approaches can indicate relative concentration, they are not directly interchangeable without additional information (e.g., the moisture content of the starting material or whether fresh vs. dried fruit was used as the baseline source). Accordingly, the observed variability and incomplete reporting primarily reflect a market transparency issue rather than a definitive assessment of regulatory compliance. Among those that did, lower extract ratios (1–10:1) were the most prevalent, with 4:1 and 10:1 being the most commonly standardised. Bilberry, elderberry, and tart cherry were represented across all concentration categories, with bilberry showing the greatest variation in extract ratios. However, extract ratios alone do not fully determine the quality or potency of botanical extracts [33]. Other critical factors, including the quality of raw materials, choice of extraction solvents, extraction duration and temperature, and the presence of excipients, may influence the final composition and bioactive content of these extracts [33,35]. This variability highlights the need for standardised reporting of pigment extraction parameters in commercial supplements to support consistent interpretation of product quality and potential efficacy.

The analysis of declared anthocyanin content revealed that only 18% of the analysed products reported anthocyanin levels, with reporting formats varying across brands. Some products expressed anthocyanin content as percentages, while others reported values in milligrams. Reported concentrations ranged from 0.1% to 36%, with bilberry-based supplements dominating the dataset. Bilberry products accounted for the majority of disclosed values and spanned all concentration ranges (0–12%, 13–24%, and 25–36%), with commonly reported levels of 25% and 36%. These findings are consistent with a report by Upton [36], which indicates that many commercial bilberry extracts are standardised to 25% anthocyanidins, corresponding to approximately 36% anthocyanins [37]. However, there is limited evidence confirming the equivalence between anthocyanidin and anthocyanin content across products. In addition, an independent study employing high-performance liquid chromatography reported a maximum anthocyanin concentration of 1017 mg per 100 g fresh weight, equivalent to approximately 1.02% anthocyanins in fresh fruit [38]. This comparison suggests that achieving anthocyanin concentrations as high as 36% likely requires extensive processing and concentration of fresh or dried plant material. Tart cherry supplements were the second most frequently reported source, with concentrations ranging from 0.1% to 10%, followed by blueberry, elderberry, and cherry, each represented by one or two products. Overall, substantial gaps in disclosure were evident, with 58% of products not reporting extract concentration and 82% not declaring anthocyanin content. Such limited transparency may hinder product comparability and interpretation of bioactivity. To address this, future work should incorporate third-party testing and chromatographic quantification (e.g., HPLC/UPLC profiling of anthocyanins) to determine how declared extract ratios relate to actual measured anthocyanin content and product-to-product variability.

Serving sizes of anthocyanin-based supplements exhibited substantial variability, ranging from <500 mg to >1500 mg across different fruit sources. While bilberry supplements displayed the widest range, often including both lower and higher serving sizes, elderberry, tart cherry, and cranberry were generally concentrated within the 500–1000 mg range, with tart cherry extending into higher dosages. Less common sources, including maqui berry, blackberry, and aronia berry, were primarily limited to <500 mg per serving. Although a standardized dosage for anthocyanin content in supplements has not been established, a study evaluating the dose–response of purified anthocyanins in healthy young adults suggested that supplementation at doses greater than 80 mg/day may provide significant antioxidant and anti-inflammatory benefits [39].

The health claims associated with anthocyanin-based supplements exhibit significant diversity across different fruit sources. In Australia, products marketed as “supplements” may fall under different regulatory pathways, including The Therapeutic Goods Administration (TGA) regulated complementary medicines and foods regulated under Food Standards Australia New Zealand (FSANZ) under Standard 1.2.7 of the Australia New Zealand Food Standards Code [40]. This standard distinguishes between (a) general-level health claims, which describe a nutrient’s effect on normal health (e.g., “antioxidants support cellular health”), and (b) high-level health claims, which link a substance to reduced disease risk (e.g., “reduces risk of UTIs”) and require FSANZ pre-approval based on robust evidence. While general-level claims can be pre-approved or self-substantiated through systematic scientific reviews, high-level claims demand stricter scrutiny. In contrast, the U.S. Food and Drug Administration (FDA) regulates dietary supplement claims under the Dietary Supplement Health and Education Act (DSHEA). Under the DSHEA, structure/function claims must be accompanied by a disclaimer stating that the claim has not been evaluated by the FDA and that the product is not intended to diagnose, treat, cure, or prevent any disease [41]. The FDA differentiates between a) structure/function claims (e.g., “supports vision health”), which describe normal bodily functions without requiring pre-approval, and b) health claims (e.g., “reduces risk of heart disease”), which require FDA review.

In this study, given that 88% of analysed supplements originate from the U.S., differences in regulatory frameworks raise concerns about how these claims align with FSANZ standards, particularly in online marketplaces. The most common claims were related to antioxidant and cellular health benefits, including anti-aging and anti-inflammatory properties. We discuss these claims in the context of the peer-reviewed evidence base; however, we did not independently assess regulatory compliance for individual products. Given the high proportion of USA-origin products, future work could compare claim wording and disclaimers on USA product pages versus Australian listings to evaluate how jurisdictional requirements shape marketing language.

Bilberry emerged as the most prominent source, with claims primarily centred on vision health, followed by antioxidant properties and cardiovascular benefits. A systematic review evaluating 12 clinical trials on bilberry anthocyanosides found weak and inconclusive evidence for improving night vision, with only one of five RCTs reporting positive effects, while all seven non-randomized, placebo-controlled trials demonstrated positive outcomes, these were often limited by significant methodological flaws [42]. Moreover, most studies included were conducted between 1960 and 1980 (*n* = 6). More recent randomized controlled trials have shown improvements in visual acuity and presbyopia (a gradual loss of the eyes’ ability to focus on nearby objects), one trial involved extracts of chokeberry, honeysuckle berry, and bilberry, while another utilized a standardized bilberry extract [43,44]. Additionally, an open-label clinical trial reported improvements in macular degeneration among adults using a commercial phytochemical extract containing bilberry [45]. It is notable, however, that these more recent studies were funded by nutraceutical industries or affiliated organizations, which may introduce potential bias.

Elderberry supplements in this study were predominantly marketed for immune support, particularly for relieving symptoms of respiratory infections such as the common cold and flu. While such claims are widespread, clinical evidence remains limited and inconclusive. A systematic review examining elderberry for viral respiratory illness reported potential benefits in reducing symptom severity and duration, but the quality of evidence was low, and findings varied across studies [46]. Notably, robust clinical evidence linking elderberry supplementation to anti-inflammatory outcomes is lacking, despite preliminary reports suggesting short-term effects on selected immune markers. These limitations, together with small sample sizes and methodological variability, suggest that while elderberry may be a safe adjunct for symptom management, the scientific basis underpinning many marketed immune and respiratory health claims remains uncertain. Further independent, well-controlled clinical studies would be valuable to substantiate these claims and guide responsible product labelling.

Tart cherry supplements were primarily marketed for joint health, muscle function, and exercise recovery, alongside antioxidant and cardiovascular benefits. Substantial evidence indicates that tart cherry consumption may confer joint-related benefits, particularly in the management of gout and the reduction of serum uric acid levels [47,48,49]. In addition, a systematic review and meta-analysis demonstrated that tart cherry supplementation can significantly improve recovery of muscle function and reduce muscle soreness following strenuous exercise. These effects are likely mediated through reductions in inflammatory markers, including C-reactive protein, and interleukin-6 (IL-6), highlighting the anti-inflammatory potential of tart cherry supplementation [50].

Cranberry supplements were mainly linked to urinary tract health. Evidence supporting the role of cranberry in the prevention and management of urinary tract infections (UTIs) is comparatively robust. Two systematic reviews provide strong support for cranberry use in UTI management: one review of 28 trials reported a 26% reduction in UTI recurrence risk among healthy women, while another review of 23 trials confirmed cranberry’s efficacy as an adjunct therapy for recurrent UTIs in susceptible populations, with no major adverse effects reported [51,52]. In addition, a Cochrane review of 10 randomized controlled trials found cranberry products to be moderately effective in reducing recurrent UTIs, particularly among women with a history of prior infections [53]. However, challenges related to participant adherence and variability in cranberry formulations were identified as factors limiting consistency of efficacy across studies.

Other fruit sources, including acai berry, sweet cherry, and blueberry, were primarily associated with health claims such as antioxidant activity, cognitive support, and vision health. Hawthorn berry supplements were specifically linked to cardiovascular health benefits, while less common sources like camu camu and maqui berry emphasized immune support and antioxidant properties. The antioxidant potential of berries is well-documented through extensive in vitro and in vivo studies [6,7,54,55,56,57]. However, the extent to which these benefits translate to commercial supplements depends on factors such as the dose, stability, and bioavailability of the bioactive compounds, which may vary considerably across products.

For stakeholders, including consumers, industry professionals, and policymakers—the primary implication is that health claims serve as a pivotal marketing strategy within Australia’s expanding nutraceutical sector, yet their application is influenced by regulatory variances and the complexities of online commerce. While many claims are self-substantiated, the strength of their scientific support varies considerably, ranging from inconclusive (e.g., outdated and industry funded bilberry studies [42]) to more robust evidence (e.g., cranberry meta-analyses [51,52]). The interplay between regulatory frameworks governed by FDA and FSANZ is particularly critical in this context. While the FDA permits structure/function claims without pre-approval (with the required DSHEA disclaimer), FSANZ requires systematic scientific reviews for self-substantiated general-level claims and pre-approval for high-level health claims. This more rigorous substantiation process may result in stricter oversight of claim validity in Australia compared with the U.S. In this setting, clearer regulatory guidance and oversight mechanisms may assist in aligning imported product claims with FSANZ requirements and reducing potential consumer confusion. This could include clearer guidance for adapting FDA-permitted claims to Australian standards, enhanced monitoring of digital retail platforms for potentially non-aligned claims under Standard 1.2.7, and expanded public resources to support consumer understanding of distinctions between general-level, structure/function, and high-level health claims across jurisdictions.

With respect to unit pricing, the highest cost was observed for bilberry supplements, reaching up to AUD 7.40 per gram, while the lowest price was recorded for tart cherry supplements at AUD 0.02 per gram. Across all fruit sources, the average price was calculated to be AUD 0.78 per gram of fruit extract. The elevated cost of bilberry supplements may be attributed to several factors, including higher reported anthocyanin content, limited geographic availability, lower yields, and more extensive processing requirements. Bilberries, also referred to as European wild blueberries, grow predominantly in the wild and are primarily sourced from northern Europe, making large-scale harvesting more challenging and potentially increasing production costs [58]. In addition, bilberries are often reported to have a superior nutrient profile compared with cultivated blueberries, particularly in terms of antioxidant capacity, largely due to their high anthocyanin content. Their documented health benefits, combined with widely promoted claims related to eye health and vision—despite limited high-quality clinical evidence supporting these claims [59]—may further contribute to premium pricing. In contrast, the substantially lower cost of tart cherry supplements likely reflects greater availability, large-scale cultivation, and comparatively simpler processing requirement [60]. Tart cherries are widely cultivated in the U.S. and Europe, with comparatively better supply chains, which may have led to a lower price per gram [61]. From a consumer perspective, the wide pricing range (A$0.02–7.40 per g) underscores the need for a cost–benefit assessment when selecting fruit extract supplements, particularly for those seeking high anthocyanin content and clinically supported health benefits.

Some limitations should be noted. This study is based on publicly available, manufacturer-provided label information and product pages and therefore describes label disclosures and marketing claims, not analytically verified composition. We did not perform independent chemical quantification or authentication, so extract ratios, anthocyanin content, and botanical identity could not be verified, and issues such as adulteration or inadequate quality control cannot be assessed from our dataset alone. Because mislabelling and adulteration have been documented in the broader dietary supplement literature [62,63,64], future work should incorporate third-party testing (e.g., quantification of anthocyanins and appropriate authentication methods) to validate label-declared content and improve market transparency. Additionally, formal inter-rater agreement metrics (e.g., Cohen’s κ) were not calculated due to the dynamic nature of product websites, where content is frequently updated or removed, precluding post hoc validation. Future studies could employ prospective dual extraction with concurrent inter-rater reliability metrics, further strengthening methodological transparency. Given that most products originated from the U.S., and claim language may differ between U.S. distributor websites and Australian listings, a systematic cross-jurisdiction comparison of claim wording and supporting evidence was beyond our scope and remains an important direction for future research to understand how marketing practices adapt (or fail to adapt) to local regulatory contexts. We also did not independently assess regulatory compliance for individual products, as this would require detailed engagement with relevant regulators. Also, product labels and online listings may change over time, therefore the dataset reflects product information as available at the time of extraction and may not represent subsequent reformulations or labelling updates.

## 5. Conclusions

The Australian market for anthocyanin-based supplements is diverse and dominated by products originating from the United States, with bilberry, elderberry, and tart cherry representing the most common fruit sources. This study identified substantial gaps in product transparency, particularly in the disclosure of extract concentration and anthocyanin content, which limit product comparability and informed consumer decision-making. Importantly, label-declared extract ratios should not be interpreted as direct indicators of anthocyanin content or bioactivity without independent verification, as formulation and processing factors may contribute to substantial variability in the final extract composition. Health claims were common across products, although the strength of supporting scientific evidence varied considerably between different anthocyanin-rich fruit sources, ranging from inconclusive to robust. Pricing also varied widely, with premium pricing not always aligned with stronger evidence of bioactive efficacy. Overall, these findings highlight the need for improved consistency in labelling and stronger alignment between health claims and scientific evidence to ensure that anthocyanins are represented and utilised as bioactive pigments in a transparent, evidence-based manner within the Australian nutraceutical market.

## Figures and Tables

**Table 1:** Inclusion and exclusion criteria used for selection of anthocyanin-based dietary supplement products in the Australian online market.

Criteria	Inclusion	Exclusion	Rationale
Botanical Source	Single anthocyanin-rich botanical (e.g., bilberry, elderberry).	Multi-fruit blends or more than 1 anthocyanin sources.	To enable source-specific comparisons (health claims, pricing, extract ratios).
Single Active Ingredient	Target fruit extract is the sole primary active ingredient.	Any additional ingredient presented as a co-primary active (e.g., highlighted in the product name/front-label claims or marketed at comparable prominence), indicating a multi-active formulation (e.g., vitamin C, zinc, probiotics, non-target botanicals).	To minimise confounding when assessing health claims against the evidence base.
Excipients & Carriers	Standard technological excipients (e.g., rice flour, silica, magnesium stearate) and inert carrier oils/waxes for softgels (e.g., olive oil, beeswax).	Formulations where the carrier or excipient acts as a competing active therapeutic agent (other bioactive extracts)	To exclude competing actives that may alter primary bioactive profile.
Delivery Format	Pre-dosed solid oral formats: Capsules, tablets, and softgels.	Loose freeze-dried powders, liquids, gummies, and lozenges.	To ensure standardized comparison of “per serving” metrics, and eliminate highly variable, user-determined dosing.
Target Phytochemical	Anthocyanins	Other than anthocyanin sources such as proanthocyanidins (e.g., grape seed extract)	Distinct chemical structures, extraction methods, and bioactivities.
Pricing Data	Standard base list price or Manufacturer’s Suggested Retail Price (MSRP).	Promotional discounts, bulk discounts, or subscription pricing.	To ensure consistent, reproducible price comparisons.

## Data Availability

The original contributions presented in this study are included in the article/Supplementary Materials. Further inquiries can be directed to the corresponding author.

## Supplementary Materials

The following supporting information can be downloaded at https://www.mdpi.com/article/10.3390/foods15060992/s1, File S1: Worked examples and assumptions for anthocyanin content standardisation..
