# Supplementary material for: Anthocyanin-Rich Pigment Supplements in the Australian Online Market: Sources, Labelling Practices, and Bioactivity Claims
Source: Foods. 2026 Mar 11;15(6):992. doi: 10.3390/foods15060992 (PMC13025483; doi:10.3390/foods15060992)
Supplement: Supplementary file 1 [file foods-15-00992-s001.zip › foods-4158738-supplementary.pdf]

Supplementary File S1

Assumption 1: Anthocyanins reported in mg per serving

When anthocyanins were reported in mg, values were converted to standardised anthocyanin % per serving using the declared extract per serving (mg) as the denominator.

Formula used:

Standardised Anthocyanin (%) per serving = [Declared anthocyanin content (mg per serving)/Declared extract (mg per serving)] × 100

Product ID	Botanical Source	Declared anthocyanin content	Unit	Declared extract (mg per serving)	Standardised Anthocyanin % per serving
1	Blueberry	12.5	mg per serving	500	2.5
2	Elderberry	14	mg per serving	100	14.0
3	Bilberry	10	mg per serving	345	2.9
9	Bilberry	15	mg per serving	60	25.0
28	Bilberry	36	mg per serving	100	36.0

Assumption 2: Anthocyanins reported as percentage

When anthocyanins were reported as percentages, values were not converted and retained as percentages.

Product ID	Botanical Source	Declared anthocyanin content	Unit	Declared extract per serving (mg)	Standardised Anthocyanin % per serving
10	Blueberry	1.5	Percentage	250	1.5

21	Tart Cherry	0.3	Percentage	465	0.3
23	Bilberry	36	Percentage	60	36.0
57	Tart Cherry	0.1	Percentage	1000	0.1
59	Cherry	0.8	Percentage	750	0.8

Assumption 3: Undisclosed (UD) anthocyanin unit

When anthocyanin content was undisclosed, values were not used for analysis.

Product ID	Botanical Source	Declared anthocyanin content	Unit	Declared extract per serving (mg)	Standardised Anthocyanin % per serving
4	Cherry	UD	UD	500	UD
5	Cranberry	UD	UD	500	UD
6	Elderberry	UD	UD	500	UD
7	Tart Cherry	UD	UD	500	UD
8	Acai berry	UD	UD	500	UD
